# Diversity, distribution and organic substrates preferences of microbial communities of a low anthropic activity cave in North-Western Romania

**DOI:** 10.3389/fmicb.2023.962452

**Published:** 2023-02-07

**Authors:** Diana Felicia Bogdan, Andreea Ionela Baricz, Iulia Chiciudean, Paul-Adrian Bulzu, Adorján Cristea, Ruxandra Năstase-Bucur, Erika Andrea Levei, Oana Cadar, Cristian Sitar, Horia Leonard Banciu, Oana Teodora Moldovan

**Affiliations:** ^1^Doctoral School of Integrative Biology, Faculty of Biology and Geology, Babeș-Bolyai University, Cluj-Napoca, Romania; ^2^Institute for Research, Development and Innovation in Applied Natural Sciences, Cluj-Napoca, Romania; ^3^Department of Molecular Biology and Biotechnology, Faculty of Biology and Geology, Babeș-Bolyai University, Cluj-Napoca, Romania; ^4^Biology Centre CAS, Institute of Hydrobiology, Department of Aquatic Microbial Ecology, Laboratory of Microbial Ecology and Evolution, Ceske Budejovice, Czechia; ^5^Emil Racovita Institute of Speleology, Cluj-Napoca Department, Cluj-Napoca, Romania; ^6^Romanian Institute of Science and Technology, Cluj-Napoca, Romania; ^7^INCDO-INOE 2000, Research Institute for Analytical Instrumentation, Cluj-Napoca, Romania; ^8^Zoological Museum, Babeș-Bolyai University, Cluj-Napoca, Romania; ^9^Centre for Systems Biology, Biodiversity and Bioresources, Faculty of Biology and Geology, Babeș-Bolyai University, Cluj-Napoca, Romania; ^10^Centro Nacional de Investigación sobre la Evolución Humana, CENIEH, Burgos, Spain

**Keywords:** microbial communities, cave ecosystems, amplicon sequencing, karst cave, community-level physiological profiles

## Abstract

**Introduction:**

Karst caves are characterized by relatively constant temperature, lack of light, high humidity, and low nutrients availability. The diversity and functionality of the microorganisms dwelling in caves micro-habitats are yet underexplored. Therefore, in-depth investigations of these ecosystems aid in enlarging our understanding of the microbial interactions and microbially driven biogeochemical cycles. Here, we aimed at evaluating the diversity, abundance, distribution, and organic substrate preferences of microbial communities from Peștera cu Apă din Valea Leșului (Leșu Cave) located in the Apuseni Mountains (North-Western Romania).

**Materials and Methods:**

To achieve this goal, we employed 16S rRNA gene amplicon sequencing and community-level physiological profiling (CLPP) paralleled by the assessment of environmental parameters of cave sediments and water.

**Results and Discussion:**

*Pseudomonadota* (synonym *Proteobacteria*) was the most prevalent phylum detected across all samples whereas the abundance detected at order level varied among sites and between water and sediment samples. Despite the general similarity at the phylum-level in Leșu Cave across the sampled area, the results obtained in this study suggest that specific sites drive bacterial community at the order-level, perhaps sustaining the enrichment of unique bacterial populations due to microenvironmental conditions. For most of the dominant orders the distribution pattern showed a positive correlation with C-sources such as putrescine, γ-amino butyric acid, and D-malic acid, while particular cases were positively correlated with polymers (Tween 40, Tween 80 and α-cyclodextrin), carbohydrates (α-D-lactose, i-erythritol, D-mannitol) and most of the carboxylic and ketonic acids. Physicochemical analysis reveals that sediments are geochemically distinct, with increased concentration of Ca, Fe, Al, Mg, Na and K, whereas water showed low nitrate concentration. Our PCA indicated the clustering of different dominant orders with Mg, As, P, Fe, and Cr. This information serves as a starting point for further studies in elucidating the links between the taxonomic and functional diversity of subterranean microbial communities.

## Introduction

Subterranean environments like caves are generally characterized by combined low temperature, high humidity, and low nutrient settings (Howarth and Moldovan, 2018). Investigations of subsurface microbial ecosystems aid in enlarging our understanding of the geomicrobial interactions of speleothems genesis and microbially driven biogeochemical cycles occurring in caves (Barton and Northup, 2007). Microorganisms adapted to cave environments feature slow metabolic and growth rates (Epure et al., 2014). Despite seemingly resource-limited condition, cave sediments could be a valuable reservoir for metabolites produced by the microbial communities inhabiting the ecosystem (Epure et al., 2014; Tomczyk-Żak and Zielenkiewicz, 2016; Rangseekaew and Pathom-Aree, 2019). Moreover, in some caves these communities could represent the primary producers that sustain the entire trophic web (Barton and Northup, 2007).

Cave microbiome generally includes *Bacteria*, *Archaea*, *Fungi* and rarely some algae and *Cyanobacteria* members. Bacterial-dominated communities are most frequently reported as colonizing the cave walls or speleothems apparently depleted in organics (Lavoie et al., 2017) and playing key roles in speleothem genesis or limestone erosion (Cuezva et al., 2009). Cave ecosystems might harbor chemotrophic-based primary production as in sulfidic Movile Cave (Sarbu et al., 1996). Microbial diversity could be shaped by geochemical composition and/or anthropic activity. Bacteria present under the oligotrophic environment of caves survive using complex metabolic pathways (Ortiz et al., 2013; De Mandal et al., 2017; Oliveira et al., 2017). Thus, despite the nutrient limitations, cave systems are inhabited by highly diverse microbial communities. Early studies revealed *Pseudomonadota*, *Acidobacteriota* (synonym *Acidobacteria*), and *Actinomycetota* (synonym *Actinobacteria*) as the most abundant phyla in addition to ‘rare’ (<1% of relative abundance) or unclassified prokaryotic lineages that were also detected (Addesso et al., 2021). A large number of caves, both show and pristine caves are found in the karst areas of the Romanian Carpathians (Onac and Goran, 2019). Information on microbial diversity within karst caves with restricted human access in the Romanian Carpathians is limited. Foregoing research on Carpathian caves focused on the ecophysiological groups relevant for paleoenvironmental studies, present in karstic springs (Bercea et al., 2019; Moldovan et al., 2020), air monitoring in show caves (Bercea et al., 2018), bat guano (Borda et al., 2014), and on active bacterial communities or bacterial culturable diversity within layers of an ice cave (Iţcuş et al., 2016; Paun et al., 2019).

Taxonomic diversity of microbial communities including uncultivated fraction can be directly derived from the 16S rRNA gene sequences. Furthermore, functional information could be predicted from 16S rRNA gene amplicon-sequencing datasets by computational methods such as phylogenetic investigation of communities by the reconstruction of unobserved states (PICRUSt; Koner et al., 2021). By combining these approaches with community-level physiological profiling (e.g., BIOLOG^®^EcoPlate™), a clearer picture of microbial diversity and metabolic potential could be drawn (Koner et al., 2021).

In this work we addressed the microbial diversity and distribution in relation to microhabitat type and carbon-source utilization patterns along Leșu Cave, a non-touristic cave located in the Romanian Carpathians. To the best of our knowledge, this is the first study to look at the relationship between taxonomic diversity and metabolic fingerprinting in low-anthropic cave environments located in the Romanian Carpathians thus contributing to a better understanding of the ecological roles of microbial communities in the trophic webs of subterranean ecosystems.

## Materials and methods

### Site description

**Figure 1:**
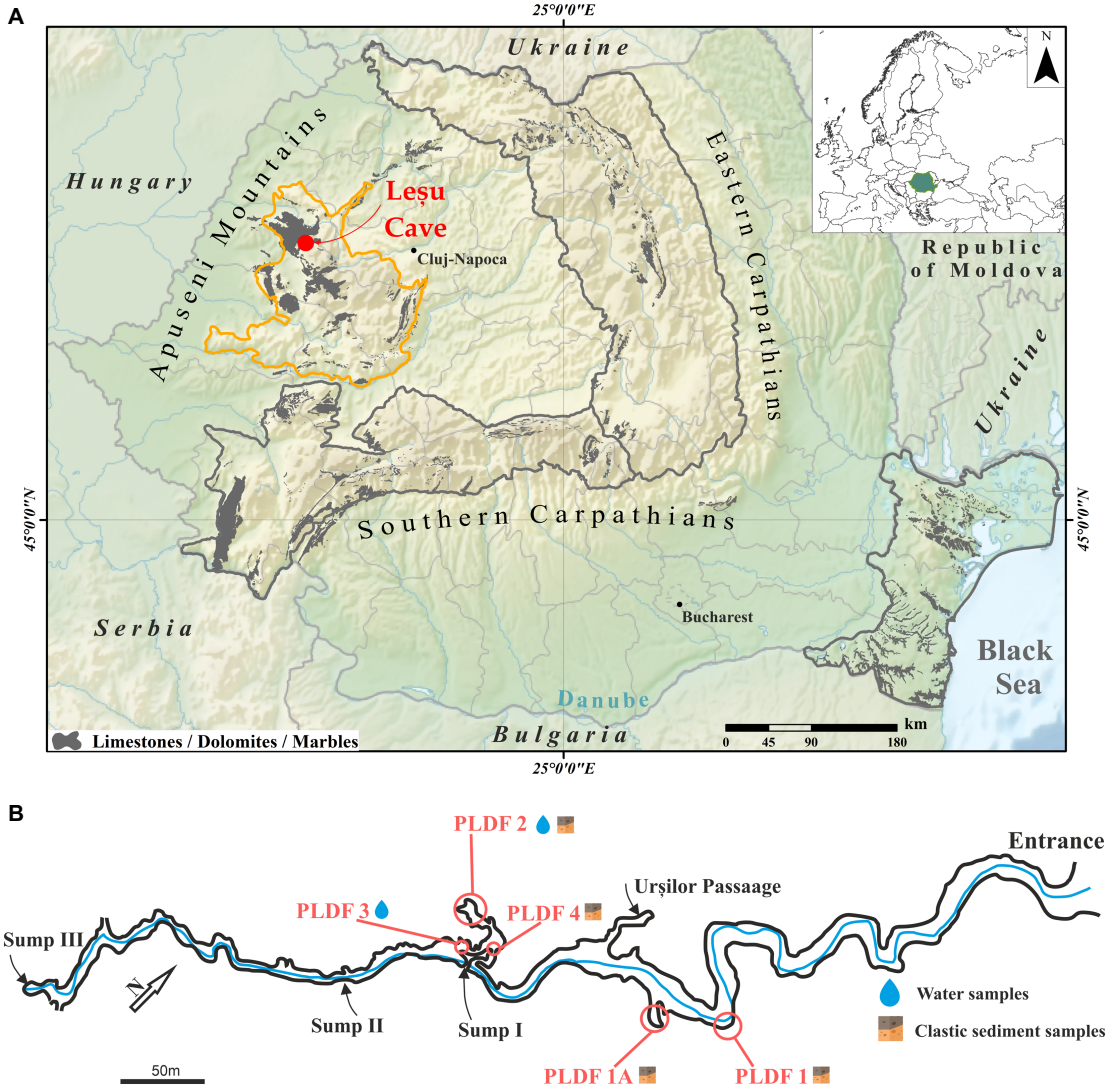
Geographical position of the Leșu Cave map (**A**; modified after Rusu, 1988) in Apuseni Mountains (NW part of Romania, maps modified in ArcGIS 10.1 from Wikipedia (https://ec.europa.eu/eurostat/web/gisco/geodata/reference-data/administrative-units-statistical-units/countries, last accessed 25th January 2023) and Eurostat (https://en.wikipedia.org/wiki/File:Relief_Map_of_Romania.png, last accessed 25th January 2023) © EuroGeographics for the administrative boundaries) with sampling sites and sample types collected **(B)**.

Peștera cu Apă din Valea Leșului (Leșu Cave; 46.8249° N, 22.5655° E, altitude 650 m a.s.l.) located in the Apuseni Mountains (Western Carpathians of Romania) is a protected area and it is categorized as a natural reserve (category IV IUCN). The cave system consists of a main gallery of approximatively 1 km crossed by a water stream with meanders for the first 300 m, forming alluvial terraces (Figures 1A, B). The annual air temperature inside the cave averages between 8.5° and 10°C. The cave is protected for the important hibernation colony with various bat species, located predominantly near the cave entrance (Zoltan and Szántó, 2003; Bücs et al., 2012).

### Sampling and sample preparation

Cave floor sediments and water samples were collected aseptically in 50 mL sterile tubes from well-established locations (Figure 1B) during February and May 2020. The six samples (Table 1) were kept at 4°C and in dark during transportation (approx. 6 h). Upon arrival in the laboratory, each sample was homogenized and subsampled for physicochemical and biological analysis. A fraction from each sample was used for metabolic fingerprinting using BIOLOG^®^ EcoPlate^™^. The plates were inoculated immediately in sterile conditions and incubated (see below), while those for chemical analysis or needing further extraction of nucleic acids were frozen at −20°C until use.

**Table 1:** Details and description of the cave samples analyzed in the present study.

Map ID	Sample ID	Sample type	Time of sampling
PLDF1	L1	Sand/clay from a terrace without direct water supply from the surface or the cave stream	Feb-20
PLDF1A	L1A	Sand/clay from a niche with relatively direct water supply from the surface, without known inlet	May-20
PLDF2	L2	Sand/clay from a lateral gallery without direct water supply from the surface or the cave stream	Feb-20
PLDF4	L4	Sand/clay from a lateral gallery without water	May-20
PLDF2W	L2W	Puddle on clay with percolation water which trickles down the wall	Feb-20
PLDF3W	L3W	Small lake under the rock with a sandy-clay substrate	Feb-20

### Physicochemical analysis

The pH and electrical conductivity (EC) were measured in water samples and 1/5 sediment to water extracts using the Seven Excellence multiparameter (Mettler Toledo, Greifensee, Switzerland). For the measurement of elements, one gram of dried sediment sample was digested with 21 mL of 12 M HCl and 7 mL of 15.8 M HNO_3_, then filtered and diluted to 100 mL with 0.5 M HNO_3_. The aqua regia-extractable fraction is the maximum amount that can be extracted, and it includes both biologically available and unavailable metals. For measurement of dissolved metals and P concentration, the water samples were acidified with 15.8 M HNO_3_ and filtered through 0.45 μm-pore size cellulose acetate membrane filters. The concentration of Na, Mg, K, Ca, P in water and of Na, Mg, K, Ca, P, Al, Fe, S, Mn in sediments was measured by inductively coupled plasma optical emission spectrometry using an Optima 5300DV (Perkin Elmer, Waltham, MA, United States) spectrometer, while the concentration of Al, Fe, As, Cr, Mn, Co, Ni, Cu, Zn in water and of As, Cr, Co, Ni, Cu, Zn in sediments was measured by inductively coupled mass spectrometry using Elan DRC II (Perkin Elmer, Waltham, MA, United States) spectrometer. The concentration of carbon, hydrogen and nitrogen in sediments was measured with a Flash 2000 CHNS/O analyzer (ThermoFisher Scientific, Waltham, MA, United States). Total nitrogen (TN) in water was measured by catalytic combustion followed by oxidation of nitrogen monoxide to nitrogen dioxide with ozone and subsequent chemiluminescence detection using a Multi N/C 2100S Analyser (Analytik Jena, Jena, Germany). Dissolved carbon (DC) and dissolved inorganic carbon (DIC) were measured in water samples filtered through 0.45 μm PTFE syringe filters by catalytic combustion and infrared detection of CO_2_ using a Multi N/C 2100S Analyser (Analytik Jena, Jena, Germany). Dissolved organic carbon (DOC) was obtained by subtracting DIC from DC. Sulphate (SO_4_^2−^), nitrate (NO_3_^−^), chloride (Cl^−^) and phosphate (PO_4_^3−^) were measured by ion chromatography on 761 Compact IC (Metrohm, Herisau, Switzerland).

### DNA extraction and amplicon sequencing for 16S rRNA genes

Total environmental DNA (eDNA) was extracted using the protocol for the Quick-DNA Fecal/Soil Microbe Miniprep Kit (Zymo Research, Irvine, CA, United States) from approximately 250 mg of each sample. The V3-V4 hypervariable regions of the prokaryotic 16S rRNA gene were amplified *via* PCR using primers 341F (5′-CCTACGGGNGGCWGCAG-3′) and 805R (5′-GACTACHVGGGTATCTAATCC-3′), according to Illumina’s 16S amplicon-based sequencing protocol. Sequencing was performed in triplicate at a commercial company (Macrogen Europe BV, Netherlands).

### Sequence analysis and comparison of microbial communities

Sequencing primers from both forward and reverse reads as well as reads containing any N characters were removed using Cutadapt v2.9 (Martin, 2011). Only reads with a minimum length of 250 nt and maximum of 301, were kept for further analysis. Paired-end reads for each sample were processed using the DADA2 package (Callahan et al., 2016) implemented in R by adapting existing pipelines.^1^ Following primer removal, paired reads were loaded into the DADA2 pipeline and trimmed (forward reads 3′ truncated at 280 nt, reverse reads 3′ truncated at 250 nt), filtered (max. 2 errors per read, minimum length after trimming = 200 nt) and finally merged with a minimum required overlap of 50 nt. Chimeras were removed from merged pairs. Following filtration and chimera removal, a total of 706,835 merged reads were retained (min per sample = 29,091, max per sample = 56,182, average = 44,177). The datasets presented in this study are publicly available at European Nucleotide Archive (ENA) under the BioProject ID PRJEB52949. Taxonomic classification of curated ASVs was achieved with the assignTaxonomy function within DADA2 against the SILVA 138 database (Pruesse et al., 2007).

Differences in community composition and statistics were computed using the phyloseq (McMurdie and Holmes, 2013) package in R and the XLSTAT (Addinsoft, Paris, France) Microsoft Excel extension (BASIC+, 2019.3.2).

Relative abundances were further generated at the genus, class, and phylum level, after performing taxonomic agglomeration using the tax glom function provided by phyloseq.

Venn analysis diagram of shared and unique ASVs was constructed using a web-based tool, InteractiVenn, developed by Heberle et al. (2015). It was used to indicate the distribution of the 16S related ASVs abundances between the different samples. TBtools was used for heatmap representations.

Bar plots of phyla and order-level were generated to show the community composition, and non-metric dimensional scaling (NMDS) was performed to determine the difference between prokaryotic community profiles on the distance matrix using Bray Curtis (Supplementary Figure S1). Shannon diversity and observed species indices were calculated using the estimate richness function from phyloseq. The Shannon index represents ASV abundance and estimates for both richness and evenness, whereas the observed species metric detected unique ASV’s present in the samples. Functional abundances were predicted based on 16S gene rRNA sequences using the picrust2_pipeline.py provided as part of PICRUSt2 (Phylogenetic Investigation of Communities by Reconstruction of Unobserved States; Parks et al., 2014). The count table of ASVs converted to biom format^2^ and ASV sequences produced with dada2 and phyloseq were used as input for the picrust2 pipeline which was run with default parameters. Full information about predicted pathways was added by employing add_descriptions.py from PICRUSt2. Statistical analyses and data representation was performed in the STAMP statistical environment (Parks et al., 2014). Microbial pathways and sample replicates were ordered by hierarchical clustering. Microbial pathways are noted by their level-2 superclasses.

Principal component analysis (PCA) of 20 physicochemical parameters with Varimax rotation and hierarchical clustering was employed using XLSTAT to interpret the structure of the principal dataset to determine which environmental variables better explain the observed bacterial community patterns in sediment samples. Due to the low number of water samples collected, and the different methods for measurements, these parameters were not included in the statistical analysis.

### Community-level physiological profile

Previous research in this cave proposed a simple and relatively quick method of screening sediment for the bacterial activity that could be used in paleoenvironmental assessment (Epure et al., 2014). Here, functional diversity within the samples was assayed using BIOLOG^®^EcoPlate^™^ (Lehman et al., 1995; Garland, 2006). Prior to inoculation on the 96-well plates, the sediment was suspended in a final volume of 50 mL of 0.85% sterile NaCl solution and then stirred at 200 rpm for 30 min. One hundred microliters of the supernatant resulted from the on-the-table sedimentation was then inoculated in each of the 96 well of BIOLOG^®^EcoPlate^™^ microplate. Water samples were directly inoculated on plates. The 96-well BIOLOG^®^EcoPlate^™^ containing 31 different carbon sources in triplicate were incubated at 25°C for 200 h and color development was monitored at 12 h using a FLUOstar^®^ Omega plate reader (BMG Labtech, Ortenberg, Germany). Parameters such as average well-color development (AWCD), substrate richness (R), Shannon-Weaver diversity index (H), and Shannon substrate evenness (E) were calculated and used to analyze the carbon source utilization pattern. For each well (i), AWCD was calculated as ∑OD_i_/31 (Garland and Mills, 1991), R was represented as the number of metabolized substrates, H = ∑p_i_(lnp_i_), where p_i_ = OD_i_ /∑OD_i_, and E was calculated as H/lnR (Garland, 2006). Average absorbance values of the triplicate reads were used, after subtracting the blank and using a 0.25 threshold value for growth response, measured at OD 590 nm. Linear correlation coefficient was calculated to establish the relationship between order-level relative abundance and absorbance values, where-1 represents a negative correlation, 0 no correlation and + 1 a positive correlation.

## Results

### Physicochemical analysis of Leșu Cave sediments and water

Most of the sediment samples were sandy or clay with or without direct water supply with increased concentrations of Ca, Fe, Al, Mg, Na and K, whereas the substrate of water samples was either sandy-clay or puddle on clay with percolation water, both with low nitrates concentration. Major elements that may influence the community structure within sediments are revealed in the PCA plot by the clustering of different dominant orders with elements such as Mg, As, P, Fe, and Cr.

Physicochemical characteristics of sediment and water samples from Leșu Cave are given in Supplementary Tables S1, S2. The pH of the sediments was slightly alkaline (8.1–8.7). Calcium (Ca) was the major element in all sediments (5–11%), followed by Fe (1.6–2.8%), Al (0.9–2%) and Mg (0.2–3.2%). The Mg content of the L1 sediment (3.2%) was one order of magnitude higher than in the other samples (0.2–0.5%). The content of As, Cr, Co, Ni, Cu, and Zn in sediments was low (<350 mg/kg) across all samples. A higher S content in sediments without direct water supply (L1) and with a relatively direct water supply (L1A, L2) was found compared to the other sediment sample (L4). The collected water sample had a typical circumneutral pH (7.9–8.1) and low electrical conductivity (170–210 μS/cm). Similar to sediments, Ca was the major element in water (41–49 mg/L), followed by Fe, Al, Mg, Na and K. The trace metal concentrations in water were below 2 μg/L in all cases. The low concentration of DOC (<1 mg/L) compared to DIC (20–24 mg/L) suggest carbonate minerals dissolution as the main source of carbon in water.

### 16S rRNA gene-based taxonomic composition of bacterial community in Leșu Cave

Following quality control and the removal of mitochondrial and chloroplast DNA sequences, a data set of 16S rRNA gene fragments (V3-V4 region) containing 706,499 clean sequences was obtained, with an average number of sequences from each sample ranging from 29,091 to 56,175. These sequences were grouped into 9,314 amplicon sequence variants (ASVs), among which ~99% and ~ 1% (36 ASVs) were assigned to *Bacteria* and *Archaea* domains, respectively.

The Chao1, Shannon, and InvSimpson indices (Table 2) were used to assess the alpha diversity of cave bacterial communities. The Shannon index was higher in the sediment samples L1A and L2 than in the other two (L1, L4) and roughly similar to the water samples. Clustering of four samples (two sediments and two water samples) on NMDS plot (Supplementary Figure S1) indicates high bacterial composition.

**Table 2:** Characteristic of diversity richness among cave sediment and water samples from Leșu Cave (R1, R2, R3 represent the number of replicates for each sample).

Sample		ASV	Chao1	Shannon	InvSimpson
L1	R1	270	271.91	4.02	25.50
	R2	283	283.60	4.13	28.71
	R3	212	212.00	3.96	22.71
L1A	R1	208	208.75	3.95	26.76
	R2	149	149.00	3.78	24.19
	R3	210	210.00	3.85	24.47
L2	R1	335	340.57	4.43	38.05
	R2	283	285.00	4.30	31.57
	R3	320	320.71	4.36	28.54
L4	R1	350	351.50	4.66	49.38
	R2	330	331.11	4.47	35.56
	R3	317	320.67	4.13	20.37
L2W	R1	216	216.00	4.06	21.69
L3W	R1	432	436.50	4.48	24.49
	R2	233	233.00	4.43	33.34
	R3	289	289.17	4.44	26.85

**Figure 2:**
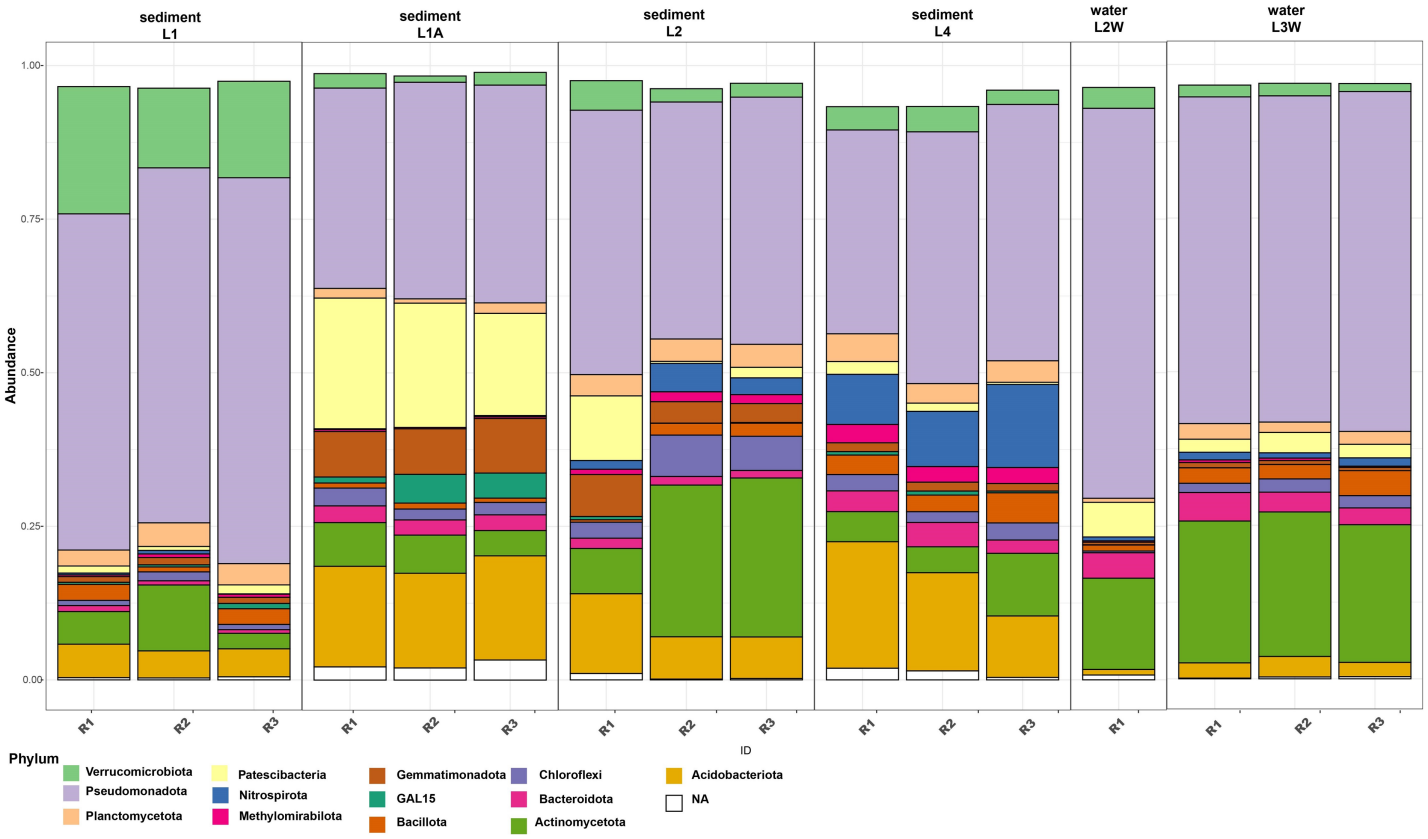
Relative abundance of dominant phyla (minimum 2% of the community) in sediments and water samples from Leșu Cave.

**Figure 3:**
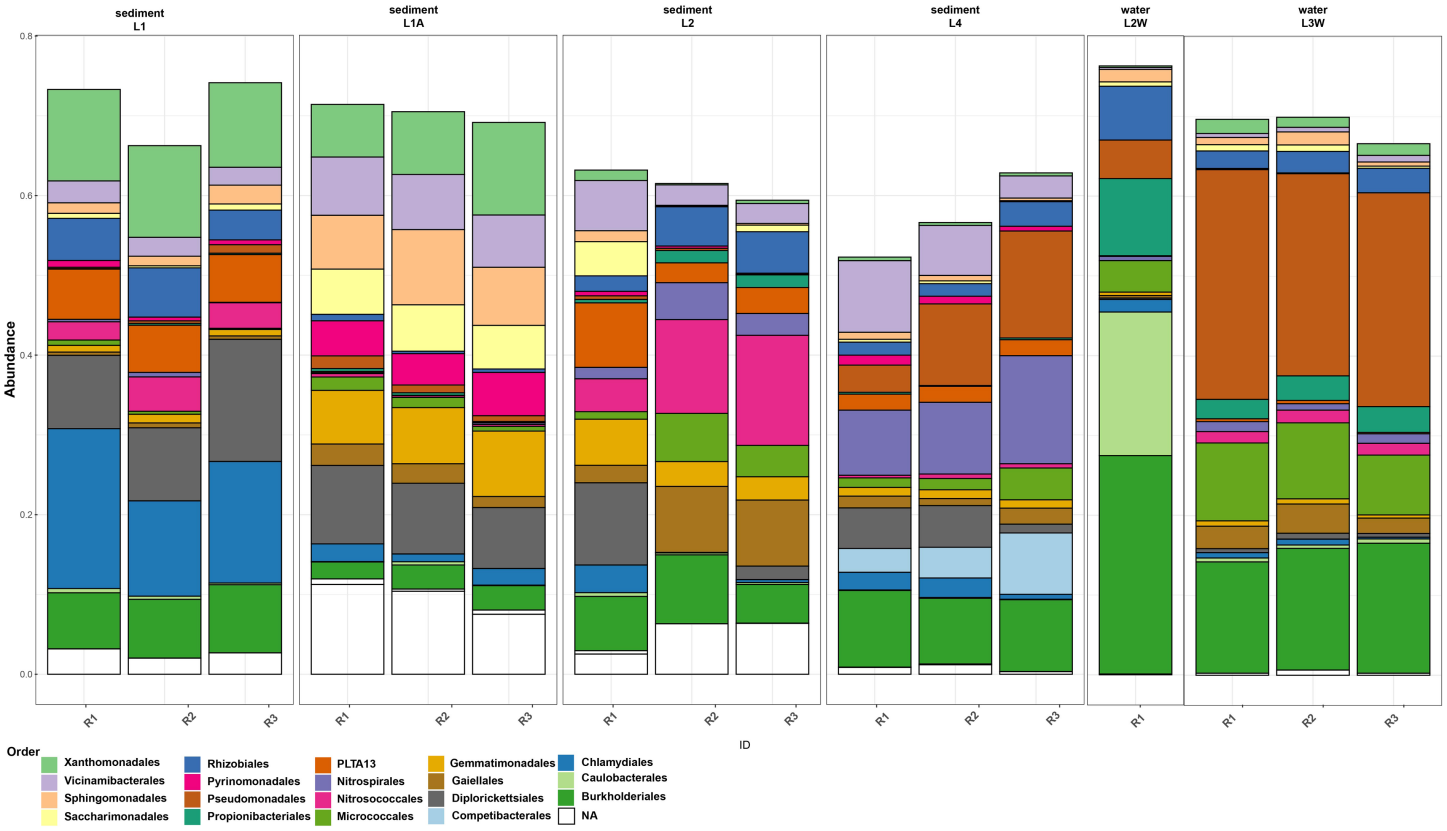
Order-level abundances of the microbial communities from sediments and water in Leșu Cave. Orders with a minimum of 5% abundance are shown.

The assessment of taxonomic composition of bacterial groups pointed a uniform distribution pattern where *Pseudomonadota* is the prevalent phylum across all samples (mean relative abundance 48.24%) followed by *Actinomycetota* (12.6%), *Acidobacteriota* (8.18%), “*Ca.* Patescibacteria” (5.68%) and *Verrucomicrobiota* (4.99%) (Figure 2). The prokaryotic communities of sediments and water in Leșu Cave were dominated by 19 bacterial orders that were prevalently present in sediment samples located closer to the cave entrance, as follows: *Chlamydiales, Diplorickettsiales*, *Xanthomonadales, Sphingomonadales*, and *Gemmatimonadales*. *Nitrosococcales*, *Nitrospirales*, and *Burkholderiales* dominated the sediments located deeper in the cave. Water samples were less diverse compared to sediments, with *Caulobacterales*, *Propionibacterales* and *Rhizobiales* highly abundant in one sample (Figure 3), whereas *Pseudomonadales*, *Gemmatimonadales*, and *Gaiellales* were dominant in the other sample. *Gamma-and Alphaproteobacteria* were the prevailing classes with relative abundances varying from 24.15–46.07% and 5.27–27.96%. Most frequently predicted orders within *Gammaproteobacteria* included *Diplorickettsiales*, *Xanthomonadales*, “*Ca.* PLTA13,” *Competibacterales* and *Burkholderiales*. “*Ca.* Patescibacteria” was found to be most abundant in L1A (19.38%) than in other samples and included “*Ca.* Saccharimonadales” and “*Ca.* Berkelbacteria” – affiliated ASVs. *Nitrospirota* was more abundant in L2 and L4 sediment samples (2.93 and 10.22%, respectively), and less abundant (below 1.2%) in water samples.

The order-level taxonomic composition (Figure 3) revealed a clumped distribution pattern where the highest abundances of *Burkholderiales* and *Pseudomonadales* were found in water samples, L2W (27.38 and 4.83% respectively) and L3W (15.16 and 26.97% respectively). In contrast, ASVs affiliated to *Gemmatimonadales, Gaiellales*, *Competibacterales*, *Nitrosococcales* and *Nitrospirales* were more abundant in sediments. Moreover, we found that “*Ca.* Saccharimonadales,” *Xanthomonadales* and *Sphingomonadales* were preferentially associated with sediment (especially L1A and L1), whereas *Caulobacterales*, *Propionibacteriales* and *Rhizobiales* related ASVs were prevalent in water sample L2W. *Micrococcales* was found to be more abundant in one water sample and two sediment samples (L2, L4, L3W), whereas “*Ca.* PLTA13” was found to be slightly more abundant in sediment samples ranging from 2.01% (L4) to 4.61% (L2) and 6.06% (L1).

**Figure 4:**
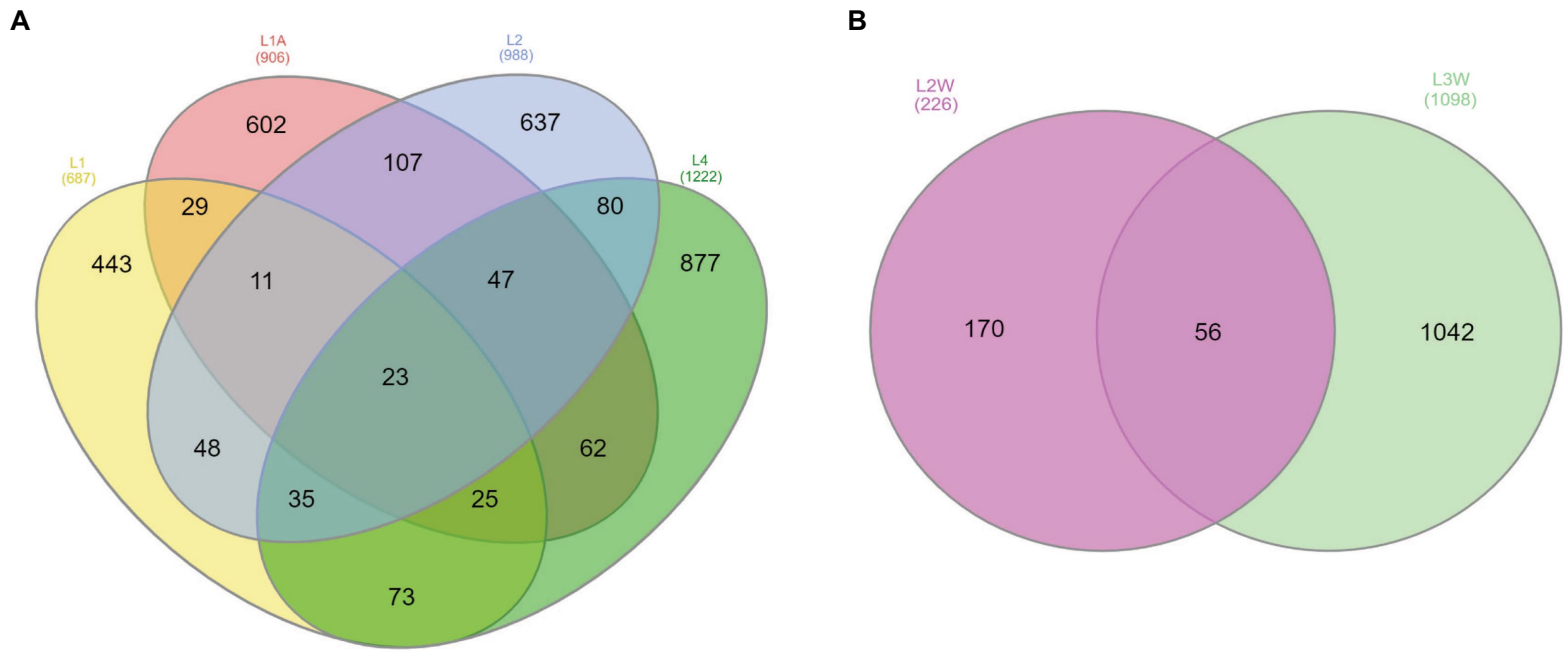
Venn diagram of the exclusive and shared ASVs at order-level with >0.1% of the community considered found among sediments **(A)** and water **(B)**.

For the Venn analysis of shared and unique ASVs, orders with >0.1% relative abundances were considered. At order level, 23 out of 3,803 ASVs were shared among sediment samples and 56 out of 1,324 for water samples (Figure 4). The sediment shared ASVs have been mainly assigned to *Rhizobiales*, *Burkholderiales*, *Vicinamibacteriales*, *Propionibacteriales*, while other unique ASVs among sediments were assigned to *Xanthomonadales*, *Spingomonadales* (genus *Sphingosinicella*), *Nitrosococcales*, and *Micrococcales*. Several unique ASVs were unassigned to order-level but assigned to *Pseudomonadota*, “*Ca.* GAL15,” and *Actinomycetota* phyla. Higher number of ASVs were shared among water samples and they were mainly classified within *Burkholderiales* (prevalent genera *Polaromonas*, *Duganella*, *Rhodoferax*), *Pseudomonadales* (predominant genera *Acinetobacter* sp. and *Pseudomonas* sp.), *Rhizobiales* (*Bradyrhizobium* sp.) and *Micrococcales* (*Arthrobacter* sp.).

### Physicochemical variables shaping the community structure

**Figure 5:**
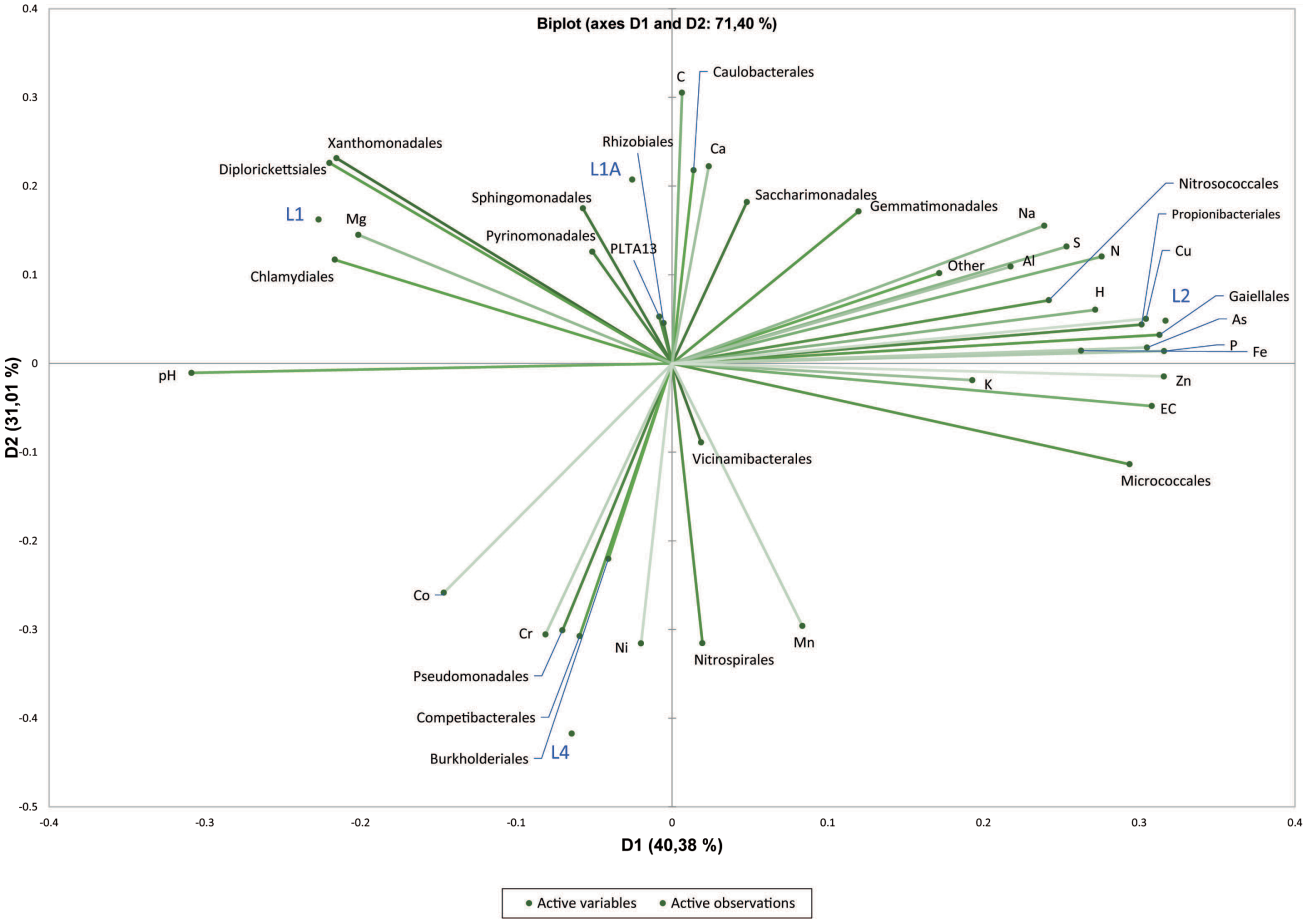
Principal Component Analysis (PCA) plot showing correlation between physicochemical factors of sediment samples with the dominant bacterial orders.

Principal component analysis (PCA) of physicochemical parameters and the relative abundances of the dominant orders was performed only for sediment samples, due to the low number of water samples. PCA indicated that, except for L1 and L1A, the sediment samples are geochemically distinct, and the first two principal components explained 71.40% of the total variance. The trace elements from sediments do not represent the actual concentration in pore water nor the trace element fraction that is readily available for bacteria but the maximum amount of trace elements that could be solubilized into the pore water under certain extreme conditions. The samples L1 and L1A were similar and grouped in the 2-dimensional PCA plot defined by Mg, *Chlamydiales, Diplorickettsiales* and *Xanthomonadales* (L1), *Sphingomondales* (L1A), whereas L2 and L4 were highly different and spatially separated on the plot. L2 is defined by *Propionibacteriales, Nitrosococcales*, Cu, As, P, Fe, whereas L4 is defined by *Competibacterales* and *Pseudomonadales*, respectively Cr (Figure 5).

### Community-level physiological profile and predicted functional pathways

**Figure 6:**
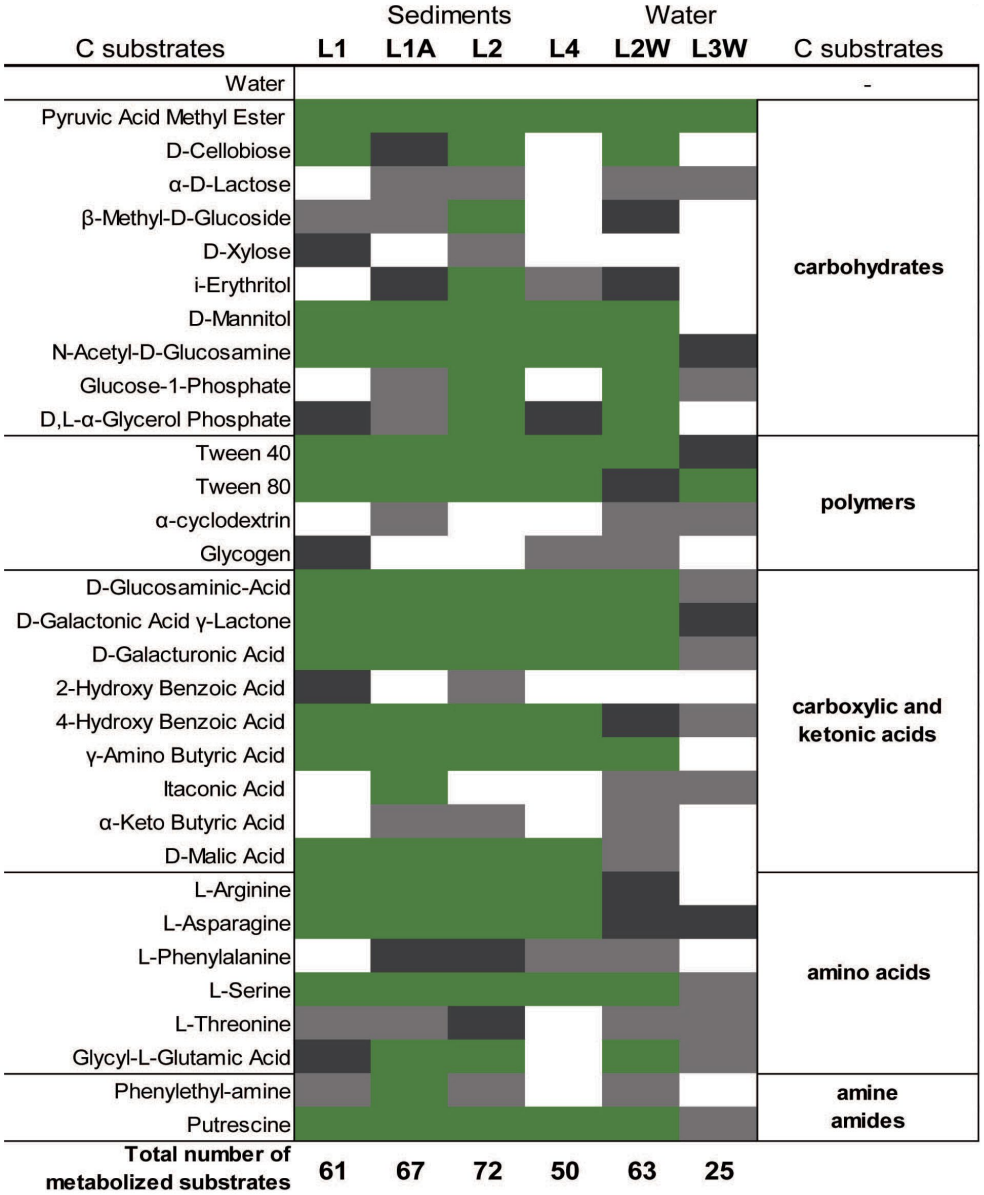
Color map of the metabolized different C-sources (substrates) among sediment and water samples collected. Substrate triplicate wells in which the color reaction occurred are marked with green (3/3 wells), dark grey (2/3 wells), light grey (1/3), and white (0/3).

Parameters such as average-well color development (AWCD), substrate richness (R) and diversity index H calculated after incubation at room temperature for 200 h using BIOLOG^®^EcoPlate^™^ indicated a higher functional diversity in sediments (substantially in L2 and L1A) than in water samples (Figure 6 and Supplementary Table S3). It was overall observed that the carbon utilization rate was higher in sediments than in water (Figure 6). Interestingly, among polymers, Tween 40 and Tween 80 were consumed in all cases whereas glycogen and 2-hydroxy benzoic acid were the least degraded.

**Figure 7:**
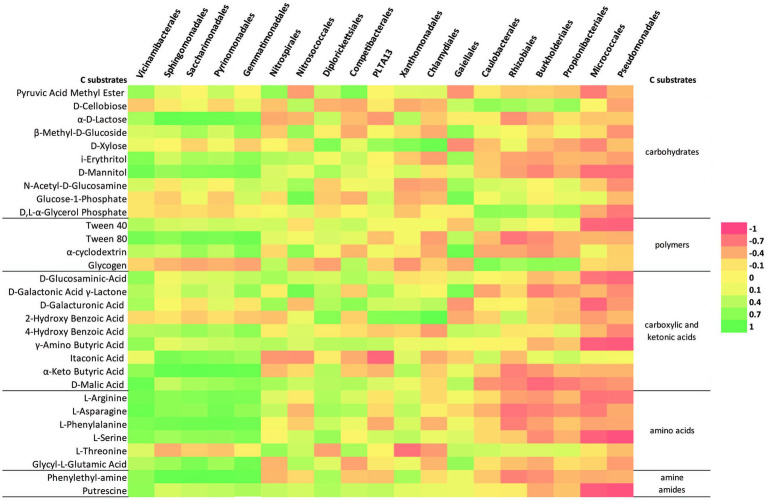
Heatmap of the correlation matrix between the relative abundance (order-level at minimum 5% of community) and C-source metabolization.

The linear correlation matrix depicting the relationship between the relative abundance of dominant order-level (minimum 5% of the community was considered) and C-source (Figure 7). With a few exceptions (*Caulobacterales*, *Rhizobiales*, *Burkholderiales*, *Propionibacteriales*, *Micrococcales*, and *Pseudomonadales*), most of the orders have a clustered distribution pattern with a positive correlation with putrescine, γ-amino butyric acid, and D-malic acid. *Sphingomonadales*, “*Ca.* Saccharimonadales*,” Vicinamibacterales*, *Pyrinomonadales*, and *Gemmatimonadales* were positively correlated with polymers (Tween 40, Tween 80, α-cyclodextrin), carbohydrates (α-D-lactose, β-Methyl-D-glucoside, i-erythritol, D-mannitol), most of the carboxylic and ketonic acids, amino acids (glycyl-L-glutamic acid, and phenylethyl-amine). Similarly, *Caulobacterales*, *Rhizobiales*, *Burkholderiales*, and *Propionibacteriales* correlated positively with D-cellobiose, D, L-α-glycerol phosphate, and glycogen. *Micrococcales* and *Pseudomonadales*, on the other hand, had a negative correlation with most of the substrates. *Nitrosococcales* and *Gaiellales* are correlated particulary with Glucose-1-phosphate. The majority of the orders have a positive correlation with L-threonine amino acid and pyruvic acid methyl ester.

**Figure 8:**
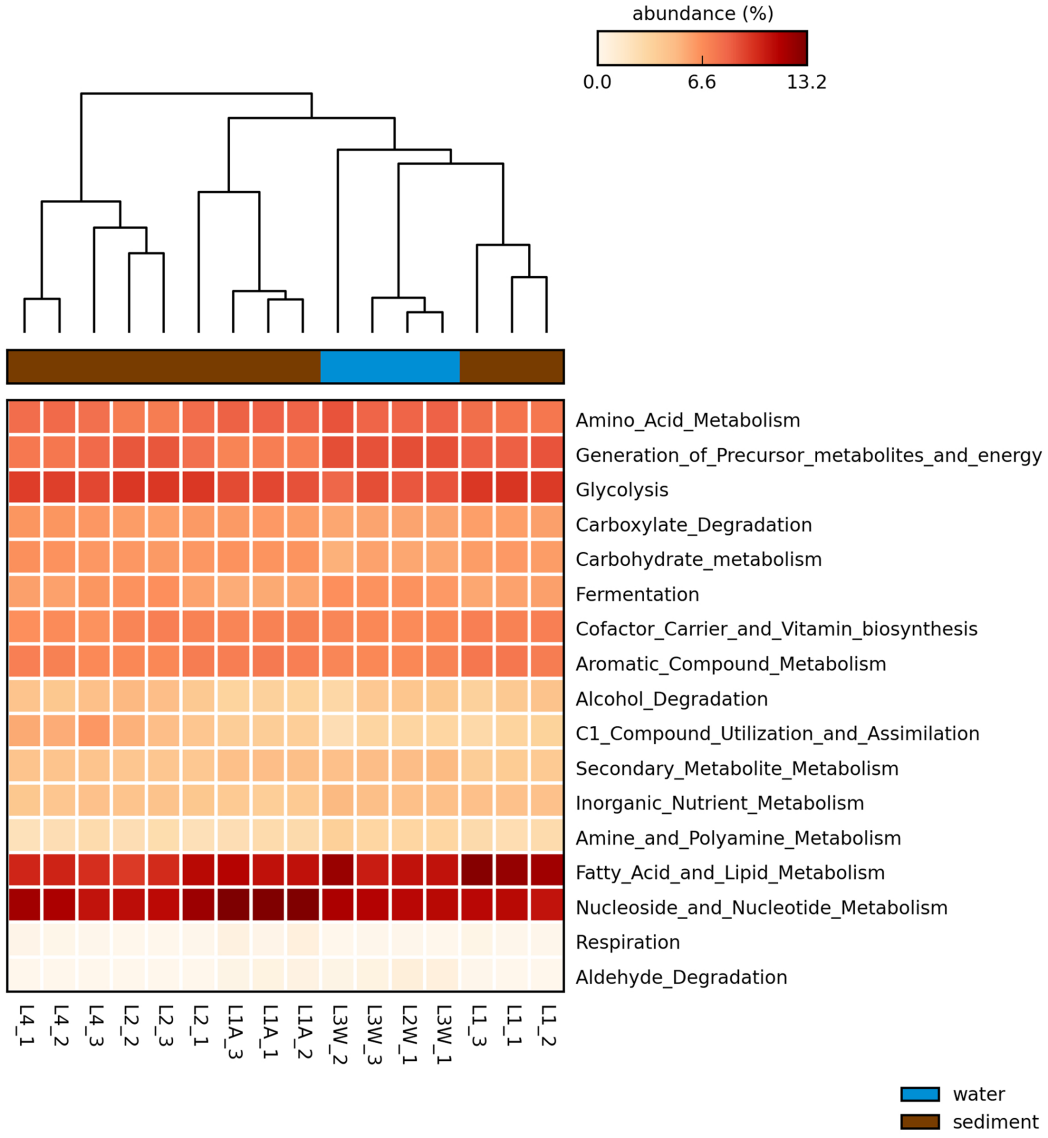
Heatmap of the proportion of sequences (5) of the predicted pathways by PICRUSt2 generated through STAMP with the average neighbor (UPGMA) method using the default parameters. Larger proportion of abundances are represented in dark red and smaller values in light yellow. Sample replicates names are included in the plot.

The PICRUSt2 analysis (Figure 8) predicted that the most abundant metabolic modules in investigated microbial communities were nucleoside and nucleotide metabolism, fatty acid and lipid metabolism, amino acid metabolism and glycolysis. The metabolic function prediction analysis also revealed enzymes involved in carbon metabolism which included alpha-amylase, pullulanase, and other carbohydrate degradation enzymes (Supplementary Tables S4–S6). Also, the analysis inferred that the enzymes involved in methane and nitrogen cycling such as methenyltetrahydrofolate cyclohydrolase (methane) and several other enzymes involved in nitrification, nitrate reduction and ammonia assimilation may be present within the microbial communities (Supplementary Tables S4–S6).

## Discussion

### Diversity and distribution patterns of bacterial communities from Leșu Cave

In this study, we profiled the diversity, distribution, and organic substrates preferences of microbial communities in a karst cave with restricted public access located in North-Western Romania. The studied Leșu Cave in NW Romania has a low anthropic impact, and therefore, its microbial communities may resemble to those inhabiting other pristine karst caves worldwide. Previous studies on bacterial communities within caves revealed that the most abundant phyla are *Pseudomonadota*, *Actinomycetota*, *Acidobacteriota*, and *Bacillota* (synonym *Firmicutes*; Wu et al., 2015; De Mandal et al., 2017). At phyla-level the distribution pattern within the cave is uniform, whereas at order-level the differences in the relative abundances depict a spatially separated distribution between sampling sites with dominant orders among sediments and water: *Chlamydiales* (L1), *Xanthomonadales* (L1A), *Nitrosococcales* (L2), *Nitrospirales* (L4), *Burkholderiales* (L2W), and *Pseudomonadales* (L3W). Interestingly, the “*Ca.* Patescibacteria*”* phylum was detected in Leșu in a single sediment sample at a relative abundance exceeding 19%. This candidate bacterial group with assumedly symbiotic lifestyle was previously found in groundwater and aquifers (Chen et al., 2021) as well as in cave sediments in a relative low abundance (Addesso et al., 2021; Chen et al., 2021). The results here show that *Pseudomonadota* had the highest relative abundance in all samples. *Alphaproteobacteria* and *Gammaproteobacteria* were the dominant classes in both sediments and water, similar to findings in Pertosa-Auletta Cave (Addesso et al., 2021), Lava Beds National Monument (Lavoie et al., 2017) or Kashmir and Tiser Cave, Pakistan (Zada et al., 2021). Predominant orders of *Alphaproteobacteria* were *Rhizobiales* and *Sphingomonadales*, whereas the *Gammaproteobacteria* orders were *Pseudomonadales*, *Nitrosococcales*, *Diplorickettsiales*, *Xanthomonadales* and “*Ca.* PLTA13.” *Aquicella* spp. (*Gammaproteobacteria*), abundant in Leșu sediments were previously reported in low abundance in Lascaux cave (Bastian et al., 2009) or other five caves from Mizoram, India (De Mandal et al., 2017). *Aquicella* spp. are presumed to be parasites or endosymbionts of amoebae (Bastian et al., 2009), suggesting an intricate trophic chain in Leșu Cave that deserves further exploration. Similarly, *Acinetobacter* spp. (*Pseudomonadales*, *Gammaproteobacteria*) was abundant in Leșu water samples. *Acinetobacter* sp. SC4, a strain involved in the precipitation of calcium carbonate (Li et al., 2019), was previously reported to be isolated from dripping waters from Altamira cave (Laiz et al., 1999), deep-well ground water (Stetzenbach et al., 1986), and other caves as well (Koren and Rosenberg, 2008; Adetutu et al., 2012; Tomova et al., 2013; Gan et al., 2020). It was suggested that the low concentrations of carbon may stimulate the growth of this bacterium and reflects its ability to utilize a variety of carbon sources and may in part explain its predominance in well water. “*Ca.* PLTA13,” a gammaproteobacterial group previously reported in the biofilm microbial community of a Mn-rich mine from Sweden (Sjöberg et al., 2020) was found in the sediments of Leșu Cave. The little information on this elusive lineage, however, prevents us from any speculation on its ecological roles within the subterranean ecosystems.

*Actinomycetota* the second most abundant phylum in this study, was frequently detected on stalactites, stalagmites, and cave walls, and as a key component of the cave soil microbiota with presumably important roles in biogeochemical cycling and weathering (Norris et al., 2011; Chen et al., 2015; Sathya et al., 2017; Zada et al., 2021). *Gaiellales* related sequences were retrieved by amplicon sequencing in this study similar to other karst (Zhu et al., 2019), and volcanic caves (Riquelme et al., 2015). The *Gaiellales* order has only one cultured representative isolated from a deep mineral water borehole that is strictly aerobic, chemoorganotrophic, being able to degrade a variety of complex substrates (Zecchin et al., 2017).

To our knowledge, this is the first evidence of *Vicinamibacterales* (*Acidobacteriota*) being reported in sediments from a karst cave with restricted access. Members of *Vicinamibacterales* are aerobic, and psychrotolerant to mesophilic chemoorganoheterotrophs, typically found in soils and growing on different simple sugars but also on complex proteinaceous compounds (Dedysh and Yilmaz, 2018). These acidobacteria are hypothesized to play an important role in soil P cycling (Wu, X. et al., 2021; Yang et al., 2022), which may explain the increased relative abundance of this *Vicinamibacteriales*-affiliated ASVs along with P concentration across a sediment sample (L2).

*Gemmatimonadales*-affiliated ASVs were recovered at low abundance in Leșu Cave sediments. This group include a few cultured members and it has been frequently detected in arid, low moisture soils, but also in cave vermiculation at low abundances (Hershey et al., 2018; Jurado et al., 2020).

We found that the geochemical composition of the sediment samples may influence the bacterial distribution among the sampling points in contrast to Alonso et al. (2018) who postulated that the mineral substrate is not the most significant factor determining the bacterial diversity among sediment and water samples. In the PCA plot, the distribution of *Sphingomonadales*, *Xanthomonadales* and *Diplorickettsiales* was influenced by Mg, which could be a key factor for bacterial growth (Cunrath and Bumann, 2019) or contribute to an increase in exopolysaccharide production and biofilm stabilization, or bacterial adhesion (Song and Leff, 2006; Wang et al., 2019).

### Community-level physiological profile and predicted functional pathways

By performing the community-level physiological profile (CLPP) approach, patterns of carbon-source utilization by microbial communities associated to Leșu Cave sediment and water were revealed. Simple carbon substrates, such as pyruvic acid methyl ester or diamine putrescine, a compound related to protein breakdown, were found to be the preferred forms of organic carbon degraded by the surveyed Leșu Cave microbial communities. The presence of *Sphingomonadales*, “*Ca.* Saccharimonadales*,” Vicinamibacterales*, *Pyrinomonadales*, and *Gemmatimonadales*, the majority of which are uncultured or poorly described lineages, was correlated with the degradation of complex polymers such as Tween 40, Tween 80, and α-cyclodextrin. In the natural settings, complex polymers feeding cave microbial communities might originate either from terrestrial environments or from cave biofilms. The latter are usually enriched in sulfated glycosaminoglycans (sGAG), negatively charged, highly hydrophilic polymers. Recently, strong evidence was brought that sGAGs-like polymers in biofilms from Sulfur Cave (Romania) can be directly produced by prokaryotes (De Bruin et al., 2022).

Interestingly, among polymers, Tween 40 and Tween 80 were consumed in all cases whereas glycogen and 2-hydroxy benzoic acid were the least degraded. Glycogen is a highly branched polysaccharide that serves as a source of readily available glucose for many organisms. Glycogen accumulates in bacterial cells during the stationary phase or during inorganic nutrient limitation (Preiss and Romeo, 1994). Glycogen degradation may be performed by a set of extracellular enzymes (i.e., glycogen phosphorylases and glycosidases such as α-amylases) specific to bacteria that complete the degradative processes to carbohydrates (Coutinho et al., 2003), which are probably less abundant in Leșu.

“*Ca.* Saccharimonadales” (“*Ca.* Patescibacteria*”*) was found to be a major contributing taxon in a biofilm on various polymers and particles from a headwater stream of wastewater treatment plants (Wu, H. et al., 2021), inferring their ability to degrade complex polymers. Besides, Tween 40 was metabolized by *Sphingomonas* sp. isolated from lake sediments of southern Finland (Rapala et al., 2005) and bacterial communities from cave ice layers (Iţcuş et al., 2016), Himalayan or Antarctic soils (Sanyal et al., 2018). Apparently, microbial communities from these low-temperature and oligotrophic habitats are readily exploiting chemically heterogeneous organic matter. Koner et al. (2021) noted that the non-ionic detergents were the fastest metabolized polymers by the rock microbiota. Utilization of carbon by bacterial communities is a key factor in the process of biomineralization in karst areas, and the efficiency of carbon catabolism is associated with the availability of different C-sources. Amino acid and carbohydrate consumption was predominantly linked to the outside-cave samples in the study of Koner et al. (2021), while other compounds were linked with the inside-cave samples, but in Leșu Cave we observed a positive correlation for the cave samples with most of the compounds. A study by Kenarova et al. (2014) showed that carbon substrates belonging to low molecular weight organic substances (LMWOS) are preferentially taken up by bacteria found in caves. Our results, highlighted by both BIOLOG^®^EcoPlate^™^ and PICRUSt analysis, showed that the bacterial communities are capable to have a moderate utilization of the carbon substrates belonging to LMWOS, such as fatty acid and lipid-related, amino acids and carbohydrates metabolic modules. Common metabolic pathways, e.g. carbohydrates and amino acids, within microbial communities were previously reported in a limestone cave in Southern Taiwan, similar to Leșu Cave, where dominating bacterial orders were positively correlated with the consumption of carbohydrates and amino acids (Koner et al., 2021). Our snapshot on the involvement in C and N cycles of bacterial communities inhabiting Leșu Cave predicted methenyltetrahydrofolate cyclohydrolase, an enzyme that drives reverse methanogenesis, similar to what has been reported by De Mandal et al. (2017) in caves from Mizoram, Northeast India. In the present study most of the genes involved in nitrogen cycle were inferred through PICRUSt analysis. The information about the role of bacteria in the nitrogen cycle in cave habitats is limited, but some reports point out their capacity to acquire energy through nitrogen cycling processes (De Mandal et al., 2017).

*Acinetobacter* sp. was one of the strains isolated from Altamira cave by Laiz et al. (1999). They further explored its ability of crystal formation when cultivated on a medium enriched with calcium and glucose. Their findings revealed that this strain is capable of producing large amounts of crystals which may help to explain why dominant orders in our study show a positive correlation with simple carbon substrates and point to the potential contribution to crystal formation inside the cave.

## Conclusion

In this study, we surveyed the molecular diversity of microbial communities associated to sediments and water in a karst cave with little anthropic impact. Sediments were geochemically distinct with higher concentrations of Ca, Fe, Al, Mg, Na, K compared to water. Nitrate concentrations in water samples were low. Overall, the studied cave microbial communities were largely dominated by *Pseudomonadota* and *Actinomycetota* phyla. The calculated diversity indices revealed substantial community richness and spatial variation. These findings are at least partly explained by a few geochemical factors (e.g., Mg, As, P, Fe, and Cr) that appear to shape the structure, composition and distribution of detected sediment and water-associated bacterial communities. The analysis of carbon substrate metabolization rates by Community-Level Physiological Profiling (CLPP) approach showed positive correlations between the most of the detected order-level taxa and the metabolization of low molecular weight C-sources such as putrescine, γ-amino butyric acid and polymers (Tween 40, Tween 80, and α-cyclodextrin). The CLPP pattern along with the predicted metabolic functions suggest that the detected bacterial communities may play key roles in the Leșu Cave trophic web by driving organic matter mineralization.

To the best of our knowledge this is the first investigation on the relationship between bacterial taxonomic diversity and organic carbon source utilization in a subterranean environment located in Eastern Europe. Nevertheless, more information on microbial activity and metabolic capabilities are yet needed to shed light on the roles of bacterial communities in the organic nutrients’ degradation and biogeochemical cycles of main elements in cave ecosystems.

## Data availability statement

The datasets presented in this study can be found in online repositories. The names of the repository/repositories and accession number(s) can be found in the article/Supplementary material.

## Author contributions

DFB, HLB, and OTM designed the research and drafted the manuscript. DFB, AIB, IC, AC, and OTM conducted the research. AIB, IC, and P-AB analyzed the bioinformatic data. OTM performed the statistical analyses. RN-B, CS, and OTM performed the sampling. EAL and OC performed the chemical analyses. All authors contributed, verified, and approved the contents of the manuscript.

## Funding

This work was supported by a grant of the Ministry of Research, Innovation and Digitization, CNCS/CCCDI – UEFISCDI, project number 2/2019 (DARKFOOD), within PNCDI III. DFB has received financial support through the project: Entrepreneurship for innovation through doctoral and postdoctoral research, POCU/380/6/13/123886 co-financed by the European Social Fund, through the Operational Program for Human Capital 2014-2020. P-AB was supported by the research grant 20-23718Y (Grant Agency of the Czech Republic).

## Conflict of interest

The authors declare that the research was conducted in the absence of any commercial or financial relationships that could be construed as a potential conflict of interest.

The reviewer FF declared a shared affiliation with the authors DFB, AIB, IC, AC, CS, and HLB to the handling editor at the time of review.

## Publisher’s note

All claims expressed in this article are solely those of the authors and do not necessarily represent those of their affiliated organizations, or those of the publisher, the editors and the reviewers. Any product that may be evaluated in this article, or claim that may be made by its manufacturer, is not guaranteed or endorsed by the publisher.

## Footnotes

^1^http://benjjneb.github.io/dada2/tutorial.html; https://f1000research.com/articles/5-1492/v2

^2^ https://biom-format.org/
