# Supplementary material for: Diversity, distribution and organic substrates preferences of microbial communities of a low anthropic activity cave in North-Western Romania
Source: Front Microbiol. 2023 Feb 7;14:962452. doi: 10.3389/fmicb.2023.962452 (PMC9941645; doi:10.3389/fmicb.2023.962452)
Supplement: Supplementary file 1 [file Data_Sheet_1.docx]

**Diversity, distribution and organic substrates preferences of microbial communities of a low anthropic activity cave in North-Western Romania**

**Authors**

Diana Felicia Bogdan^1,2🖂^, Andreea Ionela Baricz^3^, Iulia Chiciudean^3^, Paul-Adrian Bulzu^4^ , Adorjan Cristea^3^, Ruxandra Năstase-Bucur^5,6^, Erika Andrea Levei^7^, Oana Cadar^7^, Cristian Sitar^6,8^ , Horia Leonard Banciu^3,9🖂^ and Oana Teodora Moldovan^5,6,10^

^🖂^Corresponding author: D.F.B. [diana.bogdan@ubbcluj.ro](mailto:diana.bogdan@ubbcluj.ro); H.L.B. [horia.banciu@ubbcluj.ro](mailto:horia.banciu@ubbcluj.ro)

**Affiliation**

^1^ Doctoral School of Integrative Biology, Faculty of Biology and Geology, Babeș-Bolyai University, 5-7 Clinicilor Street, 400006, Cluj-Napoca, Romania

^2^ Institute for Research, Development and Innovation in Applied Natural Sciences, 30 Fântânele Street, 400327, Cluj-Napoca, Romania

^3^ Department of Molecular Biology and Biotechnology, Faculty of Biology and Geology, Babeș-Bolyai University, 5-7 Clinicilor Street, 400006, Cluj-Napoca, Romania

^4^ Biology Centre CAS, Institute of Hydrobiology, Department of Aquatic Microbial Ecology, Laboratory of Microbial Ecology and Evolution, Ceske Budejovice, Czech Republic

^5^Emil Racovita Institute of Speleology, Cluj-Napoca Department, 5 Clincilor Street, 400006, Cluj-Napoca, Romania

^6^ Romanian Institute of Science and Technology, 24-26 Saturn Street, 400504 Cluj-Napoca, Romania

^7^INCDO-INOE 2000 Research Institute for Analytical Instrumentation, 67 Donath Street, 400293, Cluj-Napoca, Romania

^8^Zoological Museum, Babeș-Bolyai University, 5-7 Clincilor Street, 400006, Cluj-Napoca, Romania

^9^Centre for Systems Biology, Biodiversity and Bioresources, Faculty of Biology and Geology, Babeș-Bolyai University, Cluj-Napoca, Romania

^10^Centro Nacional de Investigación sobre la Evolución Humana, CENIEH, 3 Paseo Sierra de Atapuerca, 09002 Burgos, Spain.

**SUPPLEMENTARY MATERIAL**

**Table S1.** Physicochemical parameters of sediment samples collected from Lesu Cave (Apuseni Mountains) during February and May 2020

| **Parameter** | **Measurement unit** | **L1** | **L1A** | **L2** | **L4** | **Median value according to GAE** |
| --- | --- | --- | --- | --- | --- | --- |
| **pH** | pH units | 8.7 | 8.6 | 8.1 | 8.6 | NA |
| **EC** | µS/cm | 53.4 | 83.9 | 115 | 83.5 | NA |
| **Na^*^** | mg/kg | 60 | 123 | 125 | 62 | 0.90 %^**^ |
| **Mg^*^** | mg/kg | 32210 | 5130 | 4300 | 2140 | 1.20 %^**^ |
| **K^*^** | mg/kg | 1250 | 2190 | 1980 | 1850 | 2.01 %^**^ |
| **Ca^*^** | mg/kg | 63800 | 111000 | 69000 | 49700 | 2.33 %^**^ |
| **P^*^** | mg/kg | 1370 | 4250 | 8930 | 3380 | 0.14 %^**^ |
| **Al^*^** | mg/kg | 9540 | 19900 | 18200 | 12200 | 10.30 %^**^ |
| **Fe^*^** | mg/kg | 16200 | 26800 | 27800 | 22300 | 1.97 % |
| **S^*^** | mg/kg | 36 | 734 | 802 | 129 | 510 |
| **Mn^*^** | mg/kg | 424 | 350 | 574 | 724 | 452 |
| **As^*^** | mg/kg | 2.3 | 16.5 | 25.7 | 10.9 | 6.0 |
| **Cr^*^** | mg/kg | 15.1 | 14.2 | 14.5 | 20.1 | 21.0 |
| **Co^*^** | mg/kg | 6.0 | 4.0 | 4.6 | 7.4 | 8.0 |
| **Ni^*^** | mg/kg | 15.3 | 14.3 | 16.4 | 24.1 | 16.0 |
| **Cu^*^** | mg/kg | 5.3 | 49.9 | 80.5 | 25.5 | 14.0 |
| **Zn** | mg/kg | 29.1 | 139 | 322 | 129 | 60.0 |
| **N** | % | 0.27 | 0.56 | 0.66 | 0.31 | NA |
| **C** | % | 3.79 | 5.04 | 3.42 | 1.12 | NA |
| **H** | % | 0.25 | 0.87 | 0.97 | 0.51 | NA |

Abbreviation use: GAE = Geochemical Atlas of Europe (<http://weppi.gtk.fi/publ/foregsatlas/article.php?id=15>); NA = Not Available; *Aqua Regia extract, ** Total concentration expressed as oxides (% - N_2_O, MgO, K_2_O, CaO, P_2_O_5_, Al_2_O_3_)

**Table S2.** Physicochemical parameters of water samples collected from Lesu Cave during February and May 2020

| **Parameter** | **Measurement unit** | **L2W** | **L3W** | **Median value according to GAE** |
| --- | --- | --- | --- | --- |
| pH | pH unit | 8.1 | 7.9 | 7.70 |
| EC | µS/cm | 171 | 209 | 30.0 |
| Na | mg/L | 0.49 | 0.49 | 6.58 |
| Mg | mg/L | 0.45 | 0.87 | 6.02 |
| K | mg/L | 0.37 | 0.48 | 1.60 |
| Ca | mg/L | 40.6 | 49.0 | 40.2 |
| P | mg/L | <0.05 | <0.05 | NA |
| Al | µg/l | 5.47 | 4.89 | 17.7 |
| Fe | µg/l | 14.7 | 16.3 | 67 |
| As | µg/l | <0.27 | <0.27 | 0.63 |
| Cr | µg/l | 1.3 | 1.6 | 0.38 |
| Mn | µg/l | <0.013 | <0.013 | 15.9 |
| Co | µg/l | <0.15 | <0.15 | 0.16 |
| Ni | µg/l | <0.13 | <0.13 | 1.91 |
| Cu | µg/l | <0.21 | <0.21 | 0.88 |
| Zn | µg/l | <0.31 | 6.9 | 2.68 |
| Cl^-^ | mg/L | 0.7 | 0.7 | 8.81 |
| NO_3_^-^ | mg/L | 4.2 | 2.3 | 2.82 |
| SO_4_^2-^ | mg/L | 4.7 | 7.7 | 16.1 |
| PO_4_^3-^ | mg/L | <0.05 | <0.05 | NA |
| TN | mg/L | 0.86 | <0.7 | NA |
| DOC | mg/L | 0.9 | 1.0 | 4.99 |
| DC | mg/L | 20.4 | 25.2 | NA |
| DIC | mg/L | 19.5 | 24.2 | NA |

Abbreviation use GAE = Geochemical Atlas of Europe (<http://weppi.gtk.fi/publ/foregsatlas/article.php?id=15>); NA = Not Available; *Aqua Regia extract

**Table S3**. Calculated parameters substrate richness (R), Shannon-Weaver diversity index (H) and Shannon evenness index (E) showing a higher functional diversity in two sediment samples (L4, L1A) and one water sample (L2W) after incubation on BIOLOG^®^ EcoPlates™

| **Sample** | **Substrate richness (R)** | ***Shannon-Weaver diversity index* (H)** | ***Shannon Eveness index* (E)** |
| --- | --- | --- | --- |
| L1 | 61 | 2.33 | 0.57 |
| L1A | 67 | 2.58 | 0.61 |
| L2 | 72 | 2.73 | 0.63 |
| L4 | 67 | 0.99 | 0.25 |
| L2W | 63 | 2.48 | 0.59 |
| L3W | 25 | 1.08 | 0.33 |


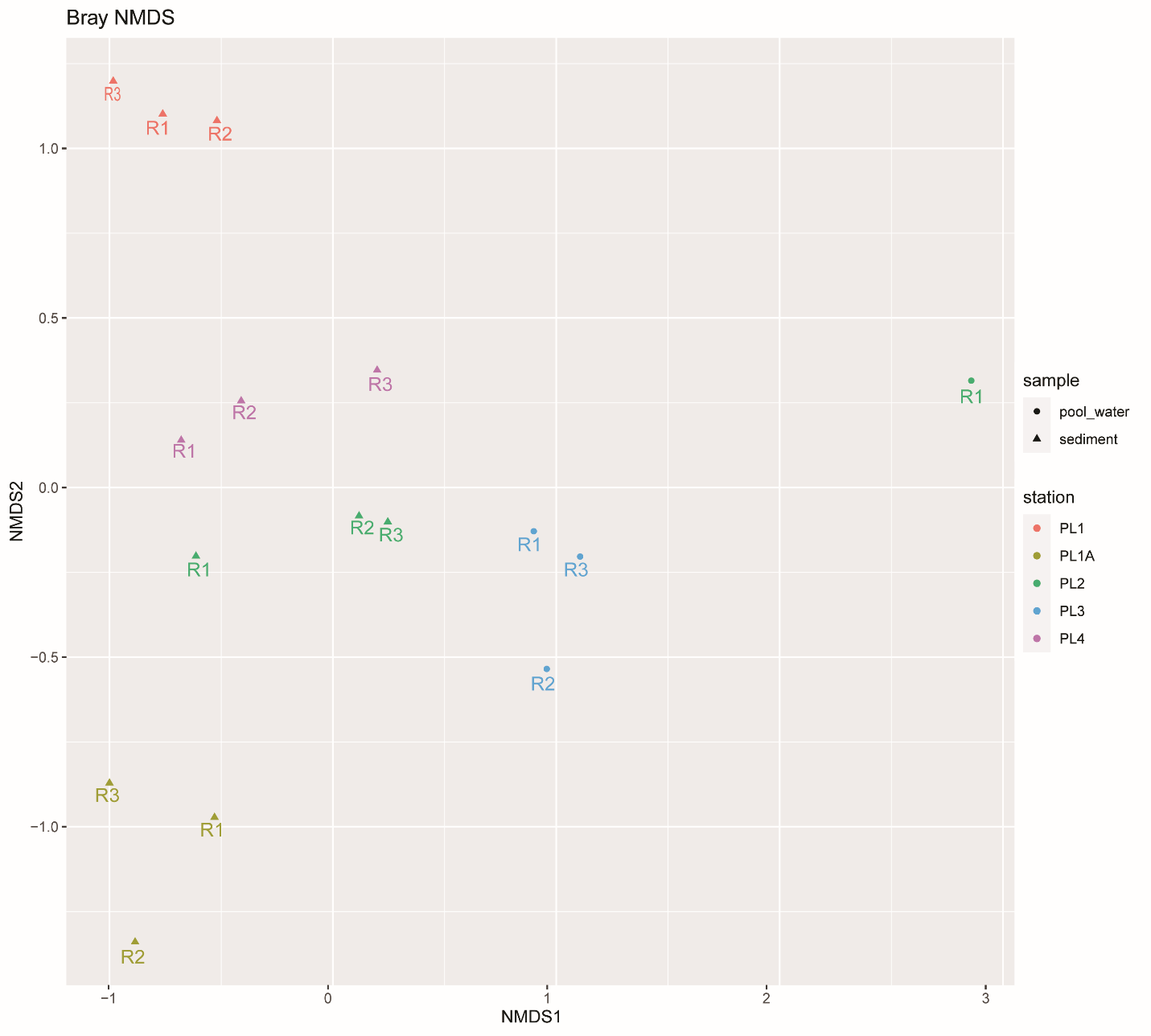


**Figure S1**. The beta diversity in the NMDS plot based on Bray dissimilarity represents a comparison of bacterial communities based on their composition (R1, R2, R3 represent the number of replicates within each sample).

**Table S4**. A selection of the genes codes for enzymes involved in carbohydrate degradation identified in cave sediments and water using PICRUST2

| **ID** | **KEGG_Description** |
| --- | --- |
| K01176 | alpha-amylase [EC:3.2.1.1] |
| K07405 | alpha-amylase [EC:3.2.1.1] |
| K01178 | glucoamylase [EC:3.2.1.3] |
| K01179 | endoglucanase [EC:3.2.1.4] |
| K01190 | beta-galactosidase [EC:3.2.1.23] |
| K05350 | beta-glucosidase [EC:3.2.1.21] |
| K05349 | beta-glucosidase [EC:3.2.1.21] |
| K01176 | alpha-amylase [EC:3.2.1.1] |
| K07405 | alpha-amylase [EC:3.2.1.1] |
| K07407 | alpha-galactosidase [EC:3.2.1.22] |
| K07406 | alpha-galactosidase [EC:3.2.1.22] |
| K01187 | alpha-glucosidase [EC:3.2.1.20] |
| K01183 | chitinase [EC:3.2.1.14] |
| K12373 | beta-hexosaminidase [EC:3.2.1.52] |
| K01191 | alpha-mannosidase [EC:3.2.1.24] |
| K01192 | beta-mannosidase [EC:3.2.1.25] |
| K01180 | endo-1,3(4)-beta-glucanase [EC:3.2.1.6] |
| K01184 | polygalacturonase [EC:3.2.1.15] |
| K01181 | endo-1,4-beta-xylanase [EC:3.2.1.8] |
| K01209 | alpha-N-arabinofuranosidase [EC:3.2.1.55] |
| K01180 | endo-1,3(4)-beta-glucanase [EC:3.2.1.6] |
| K01200 | pullulanase [EC:3.2.1.41] |

**Table S5**. A selection of the methanogenesis-associated genes identified in cave sediments and water using PICRUST2

| **ID** | **KEGG_Description** |
| --- | --- |
| **K00200** | formylmethanofuran dehydrogenase subunit A [EC:1.2.99.5] |
| **K00201** | formylmethanofuran dehydrogenase subunit B [EC:1.2.99.5] |
| **K00202** | formylmethanofuran dehydrogenase subunit C [EC:1.2.99.5] |
| **K11261** | formylmethanofuran dehydrogenase subunit E [EC:1.2.99.5] |
| **K00672** | Formylmethanofuran - tetrahydromethanopterin N-formyltransferase [EC:2.3.1.101] |
| **K01433** | formyltetrahydrofolate deformylase [EC:3.5.1.10] |
| **K01499** | methenyltetrahydromethanopterin cyclohydrolase [EC:3.5.4.27] |
| **K00320** | coenzyme F420-dependent N5, N10-methenyltetrahydro methanopterin reductase [EC:1.5.99.11] |
| **K03388** | heterodisulfide reductase subunit A [EC:1.8.98.1] |

**Table S6**. A selection of the methanogenesis-associated genes identified in cave sediments and water using PICRUST2

| **ID** | **KEGG_Description** |
| --- | --- |
| **K00459** | nitronate monooxygenase [EC:1.13.12.16] |
| **K04751** | nitrogen regulatory protein P-II |
| **K00362** | nitrite reductase (NAD(P)H) large subunit |
| **K00363** | nitrite reductase (NAD(P)H) small subunit |
| **K04488** | nitrogen fixation protein NifU and related proteins |
| **K01721** | nitrile hydratase [EC:4.2.1.84] |
| **K01501** | nitrilase [EC:3.5.5.1] |
| **K01502** | aliphatic nitrilase [EC:3.5.5.7] |
| **K05916** | nitric oxide dioxygenase [EC:1.14.12.17] |
| **K00372** | nitrate reductase catalytic subunit [EC:1.7.99.4] |
| **K00491** | nitric-oxide synthase, bacterial [EC:1.14.13.39] |
| **K00373** | nitrate reductase 1, delta subunit [EC:1.7.99.4] |
| **K00370** | nitrate reductase 1, alpha subunit [EC:1.7.99.4] |
| **K04561** | nitric oxide reductase, cytochrome b-containing subunit I |
| **K00371** | nitrate reductase 1, beta subunit [EC:1.7.99.4] |
| **K00374** | nitrate reductase 1, gamma subunit [EC:1.7.99.4] |
| **K00368** | nitrite reductase (NO-forming) [EC:1.7.2.1] |
| **K02164** | nitric-oxide reductase NorE protein [EC:1.7.99.7] |
| **K04752** | nitrogen regulatory protein P-II 2 |
| **K02586** | nitrogenase molybdenum-iron protein alpha chain |
| **K02591** | nitrogenase molybdenum-iron protein beta chain [EC:1.18.6.1] |
| **K02588** | nitrogenase iron protein NifH [EC:1.18.6.1] |
| **K04748** | nitric oxide reductase NorQ protein |
| **K02596** | nitrogen fixation protein NifX |
| **K02587** | nitrogenase molybdenum-cofactor synthesis protein NifE |
| **K02586** | nifD; nitrogenase molybdenum-iron protein alpha chain [EC:1.18.6.1] |
| **K02585** | nitrogen fixation protein NifB |
| **K02592** | nitrogenase molybdenum-iron protein NifN |
| **K02595** | nitrogen fixation protein NifW |
| **K02596** | nifX; nitrogen fixation protein NifX |
| **K02597** | nitrogen fixation protein NifZ |
| **K02593** | nitrogen fixation protein NifT |
| **K00360** | nitrate reductase (NADH) [EC:1.7.1.1] |
| **K10679** | nitroreductase / dihydropteridine reductase [EC:1.-.-.-1.5.1.34] |
| **K07218** | nitrous oxidase accessory protein |
| **K00376** | nitrous-oxide reductase [EC:1.7.99.6] |
| **K10678** | nitroreductase [EC:1.-.-.-] |
| **K02305** | nitric-oxide reductase, cytochrome c-containing subunit II |
| **K02448** | nitric-oxide reductase NorD protein [EC:1.7.99.7] |
| **K01264** | norE; nitric oxide reductase NorE protein |
| **K02598** | nitrite transporter NirC |
| **K02590** | nitrogen regulatory protein PII 2 |
| **K12265** | nitric oxide reductase FlRd-NAD(+) reductase [EC:1.18.1.-] |
| **K04747** | nitric oxide reductase NorF protein |
| **K10851** | nitrogen regulatory protein A |
